# Effects of the home-based educational intervention on health outcomes among primarily Hispanic children with asthma: a quasi-experimental study

**DOI:** 10.1186/s12889-019-7272-5

**Published:** 2019-07-09

**Authors:** Juha Baek, Ke Huang, Lucia Conner, Niko Tapangan, Xiaohui Xu, Genny Carrillo

**Affiliations:** 1 Department of Environmental and Occupational Health, School of Public Health, Texas A&M University, 212 Adriance Lab Road, College Station, TX 77843 USA; 2 Department of Statistics, Texas A&M University, Blocker Building, 3143, 155 Ireland St, College Station, TX 77843 USA; 3 Program on Asthma Research and Education, Healthy South Texas, Texas A&M School of Public Health, McAllen Campus, 2101 S. McColl Road, McAllen, TX 78503 USA; 4 Hidalgo County Health and Human Services Department, 1304 S 25th Ave, Edinburg, TX 78542 USA; 5 Department of Epidemiology and Biostatistics, School of Public Health, Texas A&M University, 212 Adriance Lab Road, College Station, TX 77843 USA

**Keywords:** Childhood asthma, Home-based asthma education, Health outcomes, Community health workers, Texas -Mexico border region, Low-income communities

## Abstract

**Background:**

Childhood asthma is a significant health issue with 8.3% prevalence in the U.S. Its prevalence is particularly higher among low-income communities in the Texas-Mexico border region, as they often lack access to clinical care and health insurance. This study examines the impact of a home-based education led by Community Health Workers (CHWs) on health outcomes for asthmatic, predominantly Hispanic children in these communities.

**Methods:**

The study was a quasi-experimental design to learn the effectiveness of the asthma home-based education by comparing changes of health outcomes between baseline and follow-up of intervention and control groups. This study enrolled 290 participants, consisting of 130 in the intervention group and 160 in the control group. The educational intervention led by the CHWs referenced the Asthma and Healthy Homes curriculum and contents of the Seven Principles of Healthy Homes. The multiple linear regression analysis was conducted to estimate the associations between the intervention and each health outcome.

**Results:**

When comparing the intervention group with the control group, the intervention group showed a significantly greater decrease in asthma attacks than the control group (*p* = 0.049). Although all of the five Children’s Health Survey for Asthma (CHSA) scores showed significant improvements between baseline and follow-up in both groups, we found that increases of CHSA scores in the intervention group were higher than the control group except for emotional health of children (EC) score. The multiple linear regression models demonstrated that the mean changes in asthma attacks (*p* = 0.036) and emotional health of families (EF) score (*p* = 0.038) were significantly better in the intervention group than the control group, adjusting for children’s age of diagnosis, household income, use of steroids, family history of allergy, and type of insurance.

**Conclusions:**

This study concluded that the home-based education by CHWs effectively improve health outcomes among children in communities lacking access to medical resources. The findings suggest the importance of the home-based education program in promoting emotional and medical care for children and their families in low-income communities like those in the Texas-Mexico border region.

## Background

In the United States, asthma is a major public health issue, with a national asthma prevalence of 8.3% in children less than 18 years old, and 10.1% in children 5–14 years old in 2016 [1]. The current asthma prevalence for Black (14.1%) and Hispanic children (7.4%) were higher than that of White children (7%) in the U.S. [2]. In a 2018 study by Buckner et al., researchers found that asthma is one of the most common chronic diseases among children, causing asthma attacks, school absence, and hospitalizations [3]. Current statistics also reveal about 54% of asthmatic children had one or more asthma attacks, nearly half of them miss more than one school day, and about 5% were hospitalized in 2016 [4]. According to a study calculating asthma-related costs, people with asthma had significantly higher total medical expenditures and school or work absences when compared to those without asthma [5]. The same report showed that costs for school or work absences and asthma-related mortality were $3 billion in 2008, and increased to $29 billion in 2013. Moreover, the estimated total cost of asthma treatment in the U.S., based on a pooled sample of 214,000 patients, was approximately $82 billion in 2013 [5].

Childhood asthma rates are high among Black and Hispanic populations, families with low educational attainment, and those who reside in low-income communities. Children in these communities are at higher risk of suffering from exposure to indoor air pollutants, such as tobacco smoke, house dust, cockroach droppings, pet dander, and mold, as well as outdoor air pollutants [6–9]. In addition, common barriers to adequate asthma care among low-income families include social, economic, literacy, and linguistic barriers [10, 11]. In addition, poorly controlled asthma contributes to frequent emergency department visits, hospital admissions, school absences, and missed workdays, all of which add a significant burden onto families [12]. Interventions to improve asthma-related outcomes for low-income children, like those living in some Texas-Mexico border communities, has centered on improving access to medical care, patient/caregiver education, and medication adherence [13, 14]. A previous study showed that families with children diagnosed with asthma normally change behaviors related to an asthma trigger, such as decluttering/cleaning the house more often and using the Integrated Pest Control Management, alongside other low-cost behavioral changes that help decrease exposure to triggers. For example, participants implemented behaviors such as “no indoor smoking” (93%), “opening windows to ventilate the home” (93%), “frequently cleaning the home” (90%), and “not leaving food uncovered or out in the open” (90%) [15].

There has been a significant increase of published research articles discussing how public health initiatives promote safe housing, and alleviate the consequences of inadequate housing. Clinicians use guidelines published by the National Asthma Education and Prevention Program (NAEPP) during their assessments of asthma severity and therapy, environmental control measures, providing education to patients, and in comprehensive pharmacologic therapy [16, 17]. Furthermore, these guidelines provide clinicians with specific information regarding how environmental triggers exacerbate asthma conditions, and how to mitigate these triggers through environmental control measures [15].

Previous studies have shown that current health education for low-income Hispanic families can reduce morbidity, hospitalizations, and use of emergency care services as well as improve asthma management skills and the quality of life for children and their caregivers [14–16]. In particular, home-based education programs providing education and training for children and parents/caregivers are widely regarded as an effective way to enhance knowledge of asthma and decrease exposure to indoor asthma triggers [13, 18]. For example, a 2015 study by Campbell et al. reported the home visit education program led by community health workers (CHWs) increased asthma patients’ symptom-free days, caregivers’ quality of life, and urgent care utilization [18].

However, there is still a paucity of studies exploring the effects of home-based asthma educational intervention in low-income Hispanic communities, including the health outcomes and healthcare utilizations of asthmatic children and their parents/caregivers. Furthermore, many studies that do regard home-based interventions only conduct pre and post-tests with one sample group, lacking any comparison sample [13, 19–21]. Therefore, the objective of this study is to examine the impact of a home-based educational intervention on asthma-related health outcomes among primarily Hispanic children in Hidalgo County, Texas, located along the Texas-Mexico border, by comparing outcome changes from baseline assessments to follow-ups, between the intervention group and a control group.

## Methods

### Participants and settings

The study used a quasi-experimental design to examine the effectiveness of the asthma home-based education intervention by comparing health outcome changes between the intervention and control groups. Eligible participants included families with children ages 1 to 17 diagnosed with asthma by a healthcare professional and living in Hidalgo County, Texas. In 2017, Hidalgo County had a population of 860,661 residents and a poverty rate of 31.2%, where 92.2% of this population identifies as Hispanic [22]. The childhood asthma prevalence for Hidalgo County was about 9.4%, higher than the national (8.3%) and state (7.6%) prevalence in 2016 [1, 9]. The research team acquired a list of schools in the county’s Independent School District (ISD) through the district’s health director, and randomly and blindly assigned the 19 total schools in this list to either an intervention group or control group. Children diagnosed with asthma attending any of the district’s schools were invited to participate in the study, with informed consent obtained from each child’s parent/guardian. Parents who did not sign the consent form provided explanations such as “My child’s health is controlled,” “I already know about asthma,” or “I do not have time to participate because I work all day.” Two community health workers (CHWs) were assigned to each intervention and control group to administer the entire study protocol, including patient management, home visits, the educational intervention, and pre/post-surveys. From May 2016 to June 2018, the education program was implemented, where two CHWs were assigned to an intervention or control group, and visited individual families at baseline and in follow-up visits every 3 months. The Institutional Review Boards of Texas A&M University reviewed and approved the study protocol.

### Intervention

The home-based asthma educational intervention was based on the Asthma and Healthy Homes curriculum certified by the Texas Department of State Health Services. The intervention aimed to teach families how to more effectively manage their child’s asthma, and create a healthier home environment. It included the following components: asthma signs and symptoms, asthma management, identifying common asthma triggers, correct use of asthma medications, emergency actions in case of asthma attacks, and fundamental components of an asthma action plan [23]. The curriculum also included content from the Seven Principles of Healthy Homes, a program developed by the National Healthy Homes Training Center and Network to reduce hazardous exposure in the household and learn how to keep the home dry, clean, ventilated, safe, pest-free, and contaminant-free [24].

The Asthma and Healthy Homes Curriculum Manual includes detailed steps for providing thorough participant training, centering the same topics included in the curriculum. Specialized instructors use comprehensive Asthma and Healthy Homes’ curriculum training modules to train Community Health Workers on how to teach the same content to the study’s participants [25]. CHW training consists of lectures, discussions, class participation/exercises, case studies, and Question & Answer sessions. After completed training, CHWs provide 60–90 min education sessions to children and families in the intervention group in either English or Spanish, utilizing the same curriculum. For the control group, CHWs only provided necessary educational materials related to asthma management without offering any direct education. CHWs provided participants in both the intervention and control groups with an allergen-proof mattress, pillow encasing, and non-chemical cleaner recipes, as well as instructions for how to use them.

### Measurements

The research team collected participants’ demographic and socioeconomic information and tracked their changing health outcomes between initial and follow-up visits using the Children’s Health Survey for Asthma, a validated and standardized tool developed by the American Academy of Pediatrics. The survey instrument conveyed health outcomes such as number of asthma attacks, hospitalizations, and emergency room (ER) visits during the last 4 weeks. The tool also asked participants to measure five health outcomes from a positive attribute of 0 (least) to 100 (most): these five health outcomes included physical health of child (PH), activities of children (AC), activities of family (AF), emotional health of children (EC), and emotional health of family (EF) [13, 26]. When measuring demographic and socioeconomic information, the survey first asked participants questions about the child participant, such as the child’s age of asthma diagnosis (continuous), the child’s gender (boy or girl), the child’s race/ethnicity (Hispanic or non-Hispanic), and if the child used inhaler steroids for more than 2 weeks (yes or no). Then, the survey focused on the family unit, focusing on family history of allergies (yes or no), household smoking (yes or no), parent/guardian’s marital status (married or non-married), household income (less than 15,000 or greater than / equal to $15,000), and type of insurance (public insurance or private insurance/self-pay).

At the baseline home visit by CHWs, all of the participants in both groups completed a pre-test and the CHSA survey. The CHW then provided 60–90 min in-person educational sessions with participants in the intervention group; those in the control group only received basic informational material with no direct education. Then, participants completed the same survey 3 months post-baseline during the follow-up visit. The changes in health outcomes from the initial and follow-up visits were compared between the intervention and the control groups.

### Statistical analysis

Descriptive statistics of the study population were calculated to estimate mean and standard deviation (SD) for continuous variables, or percentages for categorical variables. To compare differences in health outcomes between baseline and follow-up of intervention and control groups, change of values were calculated by subtracting baseline values from the follow-up values. The Pearson chi-squared tests for categorical variables and the Student t-tests for continuous variables were used to compare the baseline characteristics between participants with asthma education curriculum and those without the curriculum. Two sample t-tests were also performed to compare changes in each health outcome between intervention and control groups.

We used multiple ordinary least squares (OLS) regression models to analyze the associations between the educational intervention and change in health outcomes. Children’s age when diagnosed with asthma, use of steroids, household income, family history of allergy, and type of insurance were included in the models as confounders. With the control group, the participants without direct educational intervention, as the reference group, we calculated the point estimates and 95% confidence intervals (CIs) of the coefficient for health outcomes in association with the intervention after adjusting for confounding factors. All statistical analyses were performed by using R, version 3.5.1. A *p*-value < 0.05 was considered significant.

## Results

Of 1,272 potential participants invited by their school nurses, 313 people submitted the consent form and enrolled in the study. Of the 313 enrolled participants, the intervention group included 139 participants and the control group had 174 participants, and 130 (93.5%) and 160 (92.0%) completed the study, respectively. Table 1 shows the descriptive statistics of the study population. The average age of children when diagnosed with asthma was about 3 years old, and slightly more boys than girls participated in the study (55.5%). The majority of the children reported to be Hispanic (97.9%), and about 82% of children had a family history of allergy. About 18.4% reported to have a smoker in the household, and most of the children’s parents/guardians were married (70.3%). Nearly half of the participants (54.4%) indicated their household income is less than $15,000, and most children (90.7%) had public insurance (ex. Medicaid). About 40% of children used steroids for more than 2 weeks.

**Table 1:** Comparisons of Characteristics between Intervention and Control Groups

Variable	Total (*N* = 290) (Mean ± SD or %)	Intervention Group (*N* = 130) (Mean ± SD or %)	Control Group (*N* = 160) (Mean ± SD or %)	*p*-value
Children Age (when diagnosed with asthma)	2.99 ± 2.70	2.87 ± 2.58	3.09 ± 2.79	0.486
Children Gender				0.58
Girl	44.5%	57.7%	53.8%	
Boy	55.5%	42.3%	46.2%	
Children race/ethnicity				0.289
Hispanic	97.9%	98.4%	97.5%	
Non-Hispanic	2.1%	1.6%	2.5%	
Family history of allergy				0.625
Yes	82.2%	80.6%	83.5%	
No	17.8%	19.4%	16.5%	
Household smoker				0.493
Yes	18.4%	15.5%	20.1%	
No	81.6%	83.7%	79.9%	
Marital status of parents/guardians				0.303
Married	70.3%	73.8%	67.5%	
Non-married	29.7%	26.2%	32.5%	
Household income				0.027
Less than 15,000	54.4%	62.1%	48%	
Greater than or equal to $15,000	45.6%	37.9%	52%	
Insurance type				0.515
Public insurance	90.7%	92.3%	89.4%	
Private insurance or self-pay	9.3%	7.7%	10.6%	
Use of steroid				0.223
Yes	39.7%	35.4%	43.1%	
No	60.3%	64.6%	56.9%	

*p*-values of mean differences or differences among categories; Using two sample t-tests for continuous variables and chi-square tests for categorical variables

Table 1 compares characteristics between the intervention and control groups. Of the 290 children who completed the study, 130 children were in the intervention group while 160 were in the control group. Compared to the control group, the intervention group included fewer boys, family history of allergy, a household with a smoker, and households with a reported income of greater than or equal to $15,000. The control group had less public insurance holders and married parents/guardians than the intervention group. However, the characteristics between the two groups were not statistically different except for household income (*p* = 0.027), indicating that the two groups were comparable.

Table 2 demonstrates comparisons of health outcome variables between baseline and follow-up visits of the two groups. The number of asthma attacks decreased between baseline and follow-up in both groups, but only the difference of the intervention group was statistically significant, with a *p*-value of 0.029. A comparison of asthma attack frequency between the two groups showed that the intervention group had a significantly larger decrease than the control group (*p* = 0.049). The numbers of hospitalizations and emergency room visits displayed a modest reduction in the intervention group, while in the control group there was no change or slight increase. Moreover, the changes of hospitalizations and ER visits between baseline and follow-up between the two groups were not statistically significant.

**Table 2:** Comparisons of Health Outcome Variables between Baseline and Follow-up of Intervention and Control Groups

	Intervention group (Mean ± SD)				Control group (Mean ± SD)				*p*-value of change^c^
	Baseline	Follow-up	*p*-value^a^	Change^b^ (95% CI)	Baseline	Follow-up	*p*-value^a^	Change^b^ (95% CI)	
Number of asthma attacks	0.77 ± 2.43	0.27 ± 0.91	0.029	−0.5 (−0.86, − 0.14)	0.24 ± 0.63	0.12 ± 0.54	0.07	−0.12 (− 0.24, 0)	0.049
Hospitalizations	0.03 ± 0.28	0 ± 0	0.207	−0.03 (0.08, 0.02)	0.006 ± 0.08	0.006 ± 0.08	1	0 (−0.02,0.02)	0.235
Emergency room visits	0.05 ± 0.24	0.03 ± 0.17	0.559	−0.02 (− 0.06, 0.03)	0.05 ± 0.25	0.06 ± 0.44	0.756	0.13 (−0.06, 0.09)	0.530
PH score	72.69 ± 20.38	89.53 ± 11.76	< 0.001	16.76 (13.79, 19.73)	76.15 ± 19.08	90.74 ± 12.09	< 0.001	14.86 (12.17, 17.55)	0.351
AC score	85.26 ± 23.31	92.65 ± 15.01	0.003	7.39 (3.58, 11.21)	88.75 ± 20.84	93.87 ± 13.2	0.009	5.13 (1.95, 8.30)	0.366
AF score	85.78 ± 18.33	91.34 ± 17.32	0.013	5.5 (2.67, 8.34)	88.41 ± 16.06	91.60 ± 14.38	0.06	3.28 (1.17, 5.39)	0.215
EC score	69.23 ± 33.56	80.92 ± 26.37	0.002	11.69 (6.86, 16.52)	68.5 ± 35.06	80.31 ± 28.3	0.001	11.70 (6.92, 16.48)	0.999
EF score	62.34 ± 17	70.98 ± 13.06	< 0.001	8.63 (5.77, 11.51)	60.73 ± 18.27	66.31 ± 14.08	0.002	5.57 (3.28, 7.87)	0.1

^a^ Using two sample t test for baseline and follow-up data; ^b^ Using one sample t-tests for changes (follow-up – baseline) in each group; ^c^ Using two sample t test to compare the change results (follow-up – baseline) between intervention and control groups

*PH* physical health of children, *AC* activities of children, *AF* activities of family, *EC* emotional health of children, *EF* emotional health of families

All of the five CHSA scores showed improvements between baseline and follow-up in both groups. All of the changes in scores were statistically significant at a *p*-value of 0.05 except for AF score in the control group (*p* = 0.06). However, we found the intervention group had predominantly higher CHSA scores than the control group. The mean changes in the PH, AC, AF, and EF scores were 1.9 to 3 points higher in the intervention group than the control group. Changes in the EC score were almost identical for both groups. Nevertheless, the differences in changes between the two groups were not statistically significant.

Multiple linear regression analyses were conducted to examine the effects of a home-based educational intervention on health outcomes among Hispanic children with asthma. The results of the regression models for each health outcome are shown in Table 3. In the univariate models (Model 1), the mean change of the number of asthma attacks between baseline and follow-up was significantly lower in the intervention group than in the control group (*p* = 0.033). The regression model adjusted for children’s age when diagnosed with asthma, household income, use of steroids, family history of allergy, and type of insurance consistently showed better results in the mean changes of the number of asthma attacks (*p* = 0.036). The adjusted regression model (Model 2) demonstrated that the mean change of the EF score was significantly higher in the intervention group than the control group (*p* = 0.038). The other health outcomes did not demonstrate significant results.

**Table 3:** Results of Multiple Linear Regression Analysis

Health outcomes	Model 1 (Univariate model)			Model 2 (Adjusted model)		
	Estimate (S.E.)	95% CI	*P*-value	Estimate (S.E.)	95% CI	*P*-value
Number of asthma attacks change	−0.38 (0.18)	−0.73, −0.03	0.033	−0.40 (0.19)	− 0.76, − 0.03	0.036
Hospitalizations change	− 0.03 (0.02)	− 0.08, 0.02	0.200	− 0.03 (0.03)	− 0.09, 0.02	0.212
Emergency room visits change	−0.03 (0.05)	− 0.12, 0.06	0.555	− 0.04 (0.05)	− 0.14, 0.06	0.391
PH score change	1.90 (2.03)	−2.10, 5.89	0.351	1.26 (2.13)	−2.99, 5.51	0.560
AC score change	2.27 (2.49)	−2.63, 7.17	0.362	0.94 (2.58)	−4.14, 6.02	0.716
AF score change	2.22 (1.75)	−1.23, 5.67	0.206	1.39 (1.91)	−2.37, 5.15	0.467
EC score change	−0.01 (3.47)	−6.84, 6.83	0.999	0.97 (3.64)	−6.20, 8.14	0.790
EF score change	3.07 (1.84)	−0.55, 6.68	0.096	3.85 (2.05)	0.21, 7.49	0.038

*N* = 290; OLS regression analysis; The model 2 adjusted for children’s diagnosed age, household income, use of steroid, family history of allergy, and type of insurance

*PH* physical health of children, *AC* activities of children, *AF* activities of family, *EC* emotional health of children, *EF* emotional health of families

## Discussion

Asthma negatively affects the quality of life of children and their families because it harms their physical and emotional health, thereby influencing their daily activities. For example, asthma can cause frequent school absences or inability to participate in certain outdoor activities and sports. It may also limit family outings or even cause parents to miss work, increasing the economic and emotional burden to all family members [5, 14, 27]. The current study demonstrated that the home-based educational program for children diagnosed with asthma in disadvantaged communities reported more significant improvements in asthma attacks and asthma-related quality of life, especially in the emotional health of family (EF), when compared to the control group. This is the first study, to our knowledge, that examines the effects of home-based education for primarily Hispanic children with asthma in the Texas-Mexico border region, through a parallel group design. The study revealed the Asthma and Healthy Homes education for children and families improves their understanding of asthma signs and symptoms, and strengthens their skills to identify asthma triggers at home [28–32].

The findings of this study are consistent with those from several other studies. Our previous studies targeting Hispanic families with asthmatic children in Hidalgo County, Texas found that asthma attacks, clinic or doctor visits, CHSA scores (PH, AF, and EF), and knowledge score significantly improved after receiving home-based education through CHWs [13, 15]. A different study also reported that the Healthy Homes program (in-home environmental asthma intervention) for low-income urban households in Massachusetts decreased asthma attacks, emergency department visits, and hospitalizations, while significantly increasing all of CHSA scores [21]. However, the aforementioned studies only measure a single group’s change through the intervention, while our current study has both an intervention and control group to compare health outcomes between the two groups.

It is important to note that the current study found significant improvements in asthma attack frequency and all other CHSA scores, except for the AF score, between initial and follow-up evaluations with both groups. This study indicates home visits with basic educational materials and some allergen-proof products are still beneficial and can bring improved health outcomes, even in the absence of direct education. Nevertheless, it is evident that home-based educational intervention is more effective in improving asthma-related outcomes than home visits without direct instruction. In addition, the regression analysis results show the intervention significantly improved participants’ EF scores, when controlling several variables.

A possible explanation for this might be that the home-based instruction for at least 1 h makes parents/guardians feel cared for and emotionally supported, as they receive face-to-face interaction with experts in the comfort of their own home. Furthermore, the CHWs who led the intervention in this study are bilingual healthcare professionals with a good understanding of the target audience’s cultural norms and values [33, 34]. Consequently, CHWs can play an essential role in proficiently communicating with participants by crossing language barriers and establishing communal trust, further improving the family’s emotional health.

Meanwhile, this study demonstrates limited improvements in hospitalizations and ER visits, although the intervention group showed decreases in both areas between initial and follow-up evaluations. It would be hard to see significant changes during the short follow-up period of this study, given that hospitalizations and emergency room visits do not happen as frequently as asthma attacks do, providing a possible explanation for this result.

There are several strengths to this study. First is its study design, which is a quasi-experimental study design with a comparison group. The study also has a substantial sample size, a specific and underrepresented target population in the Texas-Mexico border region, participants’ educational involvement, and an examination of various asthma-related health outcomes. However, several limitations are worth considering when evaluating results. First, the large number of non-participants and some uncompleted participants in this study might cause a selection bias possibly. However, the selection bias may be minimized since the schools of children were randomly and blindly assigned to each group and the completion rates of both groups were high (over 90%). Second, the follow-up visit after 3 months may be too short a time to assess significant changes for some asthma-related health outcomes, such as hospitalizations and emergency room visits.

Third, this study did not deal with neighborhood factors, such as traffic-related air pollution, accessibility to healthcare, and ambient air quality, which may affect asthma-related outcomes. Future study will have to include potential neighborhood factors on the regional level. Fourth, the CHSA survey is a self-reported survey, which could provide some biases, and it only asks questions about the past 4 weeks. As such, this makes it difficult to learn about the respondents’ hospital utilization and any changes before that period. Therefore, it is advisable that a further study examines differences in health outcomes for a more extended period, based on longitudinal data. Lastly, this study did not evaluate the educational intervention’s fidelity when conducted by CHWs. A methodology for fidelity in delivering the intervention would be recommended for future study.

The findings have important implications for expanding a home-based asthma education program for children living in low-income communities, like those located along the Texas-Mexico border. Given that children and their families in under-resourced communities have limited access to asthma education, a home-visit asthma education program by CHWs could address health disparities in education and healthcare by improving child patients’ asthma control and management and reducing community asthma prevalence [35, 36]. Moreover, the study’s finding that a home-based asthma education program significantly improved the emotional health of families highlights the importance of how CHWs interact with the patients and their families by delivering instruction in-person, and in English or Spanish.

## Conclusions

The results from this study demonstrate that home-based asthma educational interventions exhibited positive results regarding asthma attack frequency and all five CHSA scores when comparing the intervention group with the control group. These results stem from families learning how to better manage their child’s asthma, identify household asthma triggers, and take preventative measures through this study’s training. In future studies, we will need to have a more extended follow-up period and use individual randomization to control personal factors, such as multiple children attending the same schools. Therefore, this study’s findings suggest expanding home-based asthma education program to low-income communities, emphasizing the importance of CHWs providing emotional support for child participants and their families.

## Data Availability

The dataset used in this study are available from the corresponding author on reasonable request.

## Abbreviations

AC: Activities of children
AF: Activities of family
CHSA: Children’s Health Survey for Asthma
CHW: Community Health Worker
EC: Emotional health of children
EF: Emotional health of families
PH: Physical health of children
